# ID1-induced p16/IL6 axis activation contributes to the resistant of hepatocellular carcinoma cells to sorafenib

**DOI:** 10.1038/s41419-018-0926-x

**Published:** 2018-08-28

**Authors:** Lei-lei Niu, Chuan-le Cheng, Ming-Yue Li, Sheng-li Yang, Bao-guang Hu, Charing C. N. Chong, Stephen L. Chan, Jianwei Ren, George G. Chen, Paul B. S. Lai

**Affiliations:** 1 Department of Surgery, Faculty of Medicine, The Chinese University of Hong Kong, Prince of Wales Hospital, Shatin, NT, Hong Kong China; 2 Department of Clinical Laboratory, The Second Hospital of Shandong University, Jinan, China; 3 Department of Thoracic Surgery, Qilu Hospital, Shandong University, 107 Wenhua Xi Road, Jinan, China; 4 Shenzhen Research Institute, The Chinese University of Hong Kong, Shenzhen, Guangdong China; 5 Cancer Center, Union Hospital, Tongji Medical College, Huazhong University of Science and Technology, Wuhan, 430022 China; 6 Department of Gastrointestinal Surgery, The Affiliated Hospital of Binzhou Medical University, Binzhou, Shandong China; 7 Department of Clinical Oncology, Faculty of Medicine, The Chinese University of Hong Kong, Prince of Wales Hospital, Shatin, NT, Hong Kong China

## Abstract

Sorafenib is the only approved drug for the treatment of advanced hepatocellular carcinoma (HCC). However, its efficacy is limited by the emergence of primary and/or acquired resistance. Senescence-associated secretory phenotype (SASP)-mediated chemo-resistance, which depends on the secreted bioactive molecules, has attracted increasing attention but never revealed in HCC. In this study, we investigated the effect of SASP-related p16/IL6 axis on sorafenib resistance in HCC. Initially, we noticed that HCC cells with a high level of p16/IL6 axis exhibited a low sensitivity to sorafenib. Further in vivo and in vitro studies demonstrated that such a primary resistance resulted from ID1-mediated activation of p16/IL6 axis. Overexpression of ID1 or IL6 blocking in sorafenib-resistant HCC cells could increase the cytotoxicity of sorafenib. Moreover, SASP-related p16/IL6 axis contributed to the formation of acquired resistance in cells received long-term exposure to sorafenib. In acquired sorafenib-resistant cells, ID1 low expression, p16/IL6 axis up-regulation, and AKT phosphorylation activation were observed. A reduced cytotoxicity of sorafenib was detected when sorafenib-sensitive cells incubated with conditioned media from the resistant cells, accompanied by the stimulation of AKT phosphorylation. The reversal of sorafenib resistance could be achieved through ID1 overexpression, IL6 blocking, and AKT pathway inhibition. Our study reveals that SASP-related p16/IL6 axis activation is responsible for sorafenib resistance, which will be a novel strategy to prevent the drug resistance.

## Introduction

Senescence is defined as a state of cell cycle arrest and can be triggered by either the sequential loss of telomeres or numerous forms of cellular stress, for example, UV irradiation, oxidative stress, or aberrant oncogenic signaling^1^. p16/CDK/pRb is one of the most studied pathways responsible for the regulation of cellular senescence^2^. It has been documented that pRb is at the core of senescence due to its repression on transcription of genes necessary for G1–S phase transition and DNA replication^3^. p16 is an important inducer of senescence, which can bind to CDK4 and inhibit its kinase activity, leading to the prevention of Rb phosphorylation^3^.

Initially, senescence was considered to be a tumor-suppressive mechanism. However, the detrimental effects of senescent cells on cancer treatment have been described in recent years^4^. Accumulating evidence demonstrated that senescent cells still appear to be metabolically active. They can secret numerous bioactive molecules, such as pro-inflammatory cytokines, chemokines, and growth factors. This phenomenon is termed as senescence-associated secretory phenotype (SASP)^5^.

Regarding cancer initiation and maintenance, both detrimental and beneficial effects of SASP have been reported. Some studies have proved that the components of the SASP can induce apoptosis of cancer cells^5^. In contrast to its anti-tumor activity, SASP have also been shown to exert pro-tumorigenic effects^6^. As a typical biomarker of SASP, IL6 can activate immune responses, leading to improved clearance of senescent tumor cells, and stimulate proliferation of neighboring tumor cells^7^.

Nowadays, chemotherapy-resistance remains a major obstacle to successful cancer treatment^8^. Sorafenib is the only clinically approved drug for the treatment of advanced hepatocellular carcinoma (HCC)^9^. However, although it exerts positive effects on overall survival, the responsiveness among HCC patients is very low. More importantly, most patients who are initially sensitive to sorafenib will ultimately develop drug resistance^10^. Therefore, understanding the mechanisms of how such chemo-resistance is generated is clinically critical.  As previously reported, cellular senescence can be inhibited by inhibitors of differentiation 1 (ID1) due to its negative effects on p16 expression. The transcription factors, Ets1 and Ets2, activate p16 expression via binding to the ETS-binding site of p16 promoter to stimulate its transcription. This activation can be prevented by a direct interaction between ID1 and Ets 1/2^11^. Another research group discovered that ID1 could transcriptionally repress p16 expression to induce cellular senescence^12^. Several studies have reported that ID1 contributes to chemo-resistance. For example, in prostate cancer, patients with ID1 up-regulation were found to be associated with a significant delay in developing biochemical relapse and ID1 overexpression could sensitize cells to docetaxel-induced cytotoxicity^13^. Lung cancer cells with high ID1 protein expression were vulnerable to the treatment of paclitaxel and cisplatin^14^. In our study, we aimed to reveal how ID1/p16-induced senescence affected the outcome of sorafenib treatment in HCC.

## Results

### Cellular senescence is positively correlated with sorafenib resistance in HCC

**Fig. 1:**
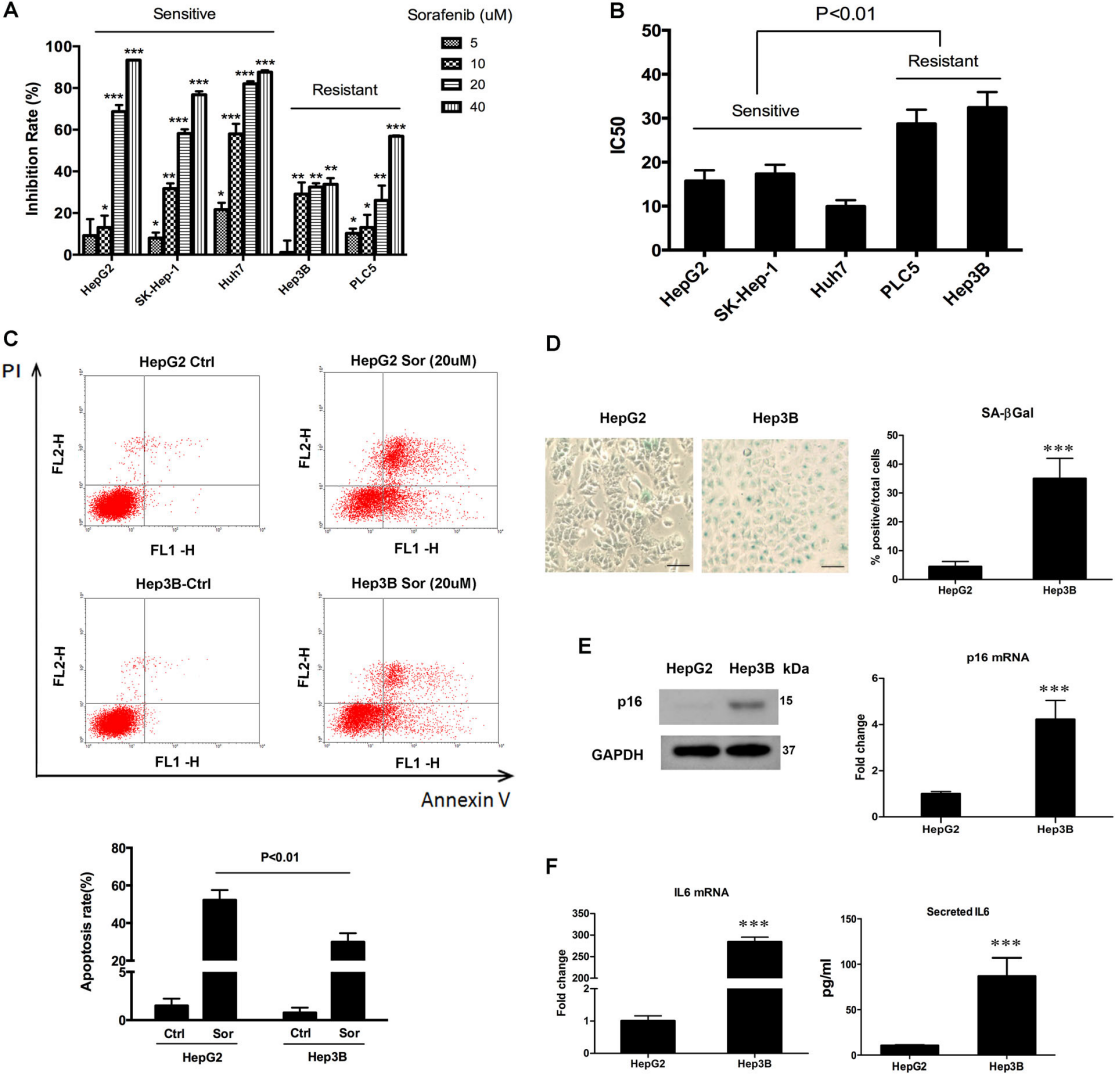
Relationship between sorafenib efficacy and cell senescence. **a** The sensitivity of five HCC cell lines to sorafenib was measured by MTT assay. Cells were seeded in 96-well plates. After overnight incubation, cells were treated with different concentrations of sorafenib (5, 10, 20, 40 μM) for 24 h, then MTT assay was performed. DMSO was used as the control. The results were shown as inhibition rate, which indicates the percentage of cell growth inhibition caused by sorafenib treatment. **b** IC50 value was calculated based on the MTT results. The value represents the drug concentration of inducing 50% growth suppression compared to control cells. **c** HepG2 and Hep3B cells were incubated with sorafenib for 24 h, cell apoptosis was evaluated through flow cytometry (upper). The apoptosis ratio was calculated as the early apoptosis (lower right quadrant) plus the late apoptosis (upper right quadrant) percentage (lower). **d** HepG2 and Hep3B cells were incubated with β-gal staining solution. Senescent cells exhibited blue staining (left). Percentages of SA-β-gal positive cells were calculated and exhibited as a histogram (right). **e** Expression level of p16 in HepG2 and Hep3B cell lines was tested by western blot (left) and qRT-PCR (right). **f** IL6 expression in HepG2 and Hep3B cell lines was measured by qRT-PCR (left) and ELISA (right). For ELISA experiment, cells were seeded in 6-well plates and cultured for 24 h, then cell supernatants were collected. *p < 0.05; **p < 0.01; ***p < 0.001, compared with relevant controls (Ctrl) or HepG2. The scale bars represent 25 μm. All immunoblots indicate molecular size markers in kDa

Initially, we observed that HCC cell lines exhibited different sensitivities to sorafenib, based on the MTT results (Fig. 1a). The IC50 (half maximal inhibitory concentration) value ranged from 9.9 μM in Huh7 to 32.4 μM in Hep3B (Fig. 1b). Cells that exhibited higher IC50 value were defined as resistant. To determine whether cellular senescence was associated with the efficacy of sorafenib, we selected the sorafenib-sensitive cell line HepG2 and sorafenib-resistant cell line Hep3B for further tests. Flow cytometry assay of cell apoptosis was conducted to confirm the MTT results. Cell apoptosis rate was different between HepG2 cells (52.26%) and Hep3B cells (29.91%) (Fig. 1c). The senescent state is characterized by induction of acidic senescence-associated-beta-galactosidase (SA-β-gal) activity^15,16^. The two cell lines were stained for SA-β-gal activity. We found that the number of SA-β-gal positive stained cells was higher in Hep3B than in HepG2 (Fig. 1d). The expression of senescence inducer p16 was higher in Hep3B than in HepG2, at both protein and mRNA levels (Fig. 1e). The mRNA level of SASP marker IL6 was increased in Hep3B cells (Fig. 1f, left). Also, we noticed that the sorafenib-resistant cell line Hep3B secreted a high level of IL6 protein into its supernatant (Fig. 1f, right).

### Senescence induction decreases the efficacy of sorafenib in HCC sorafenib-sensitive cell line

**Fig. 2:**
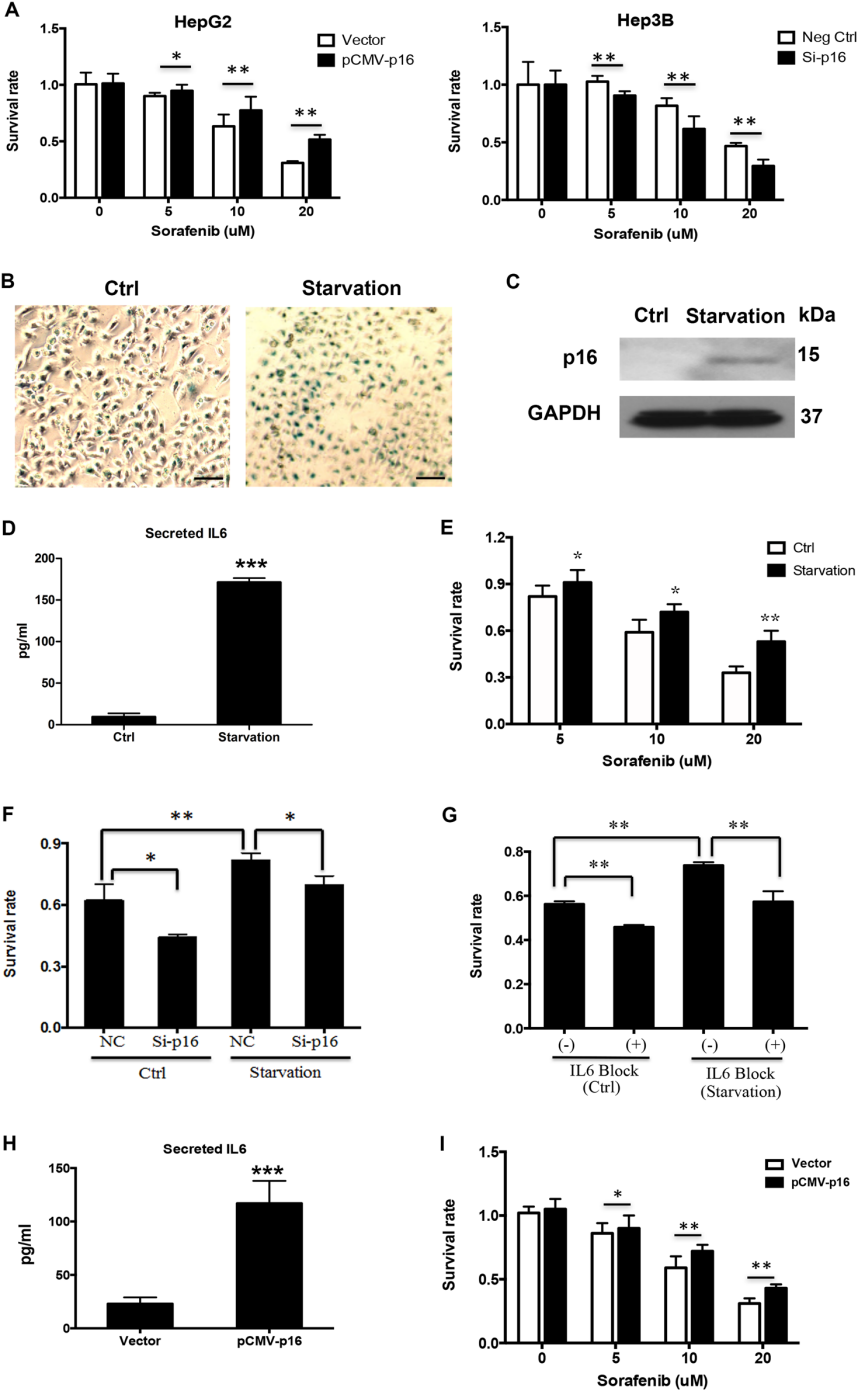
Regulation of p16 and IL6 on the inhibitory effect of sorafenib on cell viability in HCC. **a** HepG2 and Hep3B cells were transfected with p16 overexpression vector and siRNA targeting p16, respectively. After 24 h, cells were incubated with different concentrations of sorafenib for 24 h. MTT assay was conducted to test cell viability. **b** After cultured with normal medium (Ctrl) or serum-free medium (Starvation) for 24 h, HepG2 cells were stained with β-gal solution. In Starvation group, senescent cells that exhibited blue staining were frequently observed under microscope. **c** p16 expression was detected by western blot. **d** IL6 concentration in cell supernatant was measured by ELISA. **e** MTT assay was used to determine the different effects of sorafenib on cell viability in HepG2 with routine culture (control group) or serum depletion (starvation group). **f**, **g** After cultured with normal medium (Ctrl) or serum-free medium (Starvation), cells were transfected with siRNA targeting p16 for 24 h (**f**) or pretreated with IL6 neutralizing antibody (5 ng/ml) for 2 h (**g**), and then incubated with 10 μM sorafenib for 24 h. MTT assay was conducted to test cell viability. NC: negative control. **h** In a transwell co-culture system, parent HepG2 cells were seeded in upper chamber, and the cells transfected with pCMV-p16 or empty vector were seeded in the bottom of wells. After 48 h-co-culture, the supernatant in bottom cells was collected to detect IL6 concentration. **i** The parent HepG2 cells in upper chamber were incubated with different concentrations of sorafenib for 24 h. Then cell viability was tested by MTT. *p < 0.05; **p < 0.01; ***p < 0.001, compared with control (Ctrl). The scale bars represent 25 μm. All immunoblots indicate molecular size markers in kDa

According to the western blot result shown in Fig. 1e, we transfected p16 overexpression vector into HepG2 and siRNA against p16 into Hep3B (Fig. S1A). As indicated in Fig. 2a, p16 up-regulation in HepG2 reduced the sorafenib-mediated inhibition on cell viability, however, Hep3B cells with p16 knockdown became sensitive to sorafenib.

Above data implied that cellular senescence plays a positive role for sorafenib resistance. To verify the result, we induced senescence in sorafenib-sensitive cell line HepG2 through serum starvation in 0.1% FBS for 24 h, and then detected its response to sorafenib. After nutrient deprivation, cellular senescence was induced, as indicated by the frequently observed SA-β-gal positive stained cells (Fig. 2b), activation of p16 (Fig. 2c), and up-regulation of IL6 levels (Fig. 2d). As expected, HepG2 cells with serum starvation became resistant to sorafenib (Fig. 2e), and p16 knockdown could facilitate HepG2 cells to overcome the starvation-induced chemo-resistance to sorafenib (Fig. 2f). Considering that IL6 is an important SASP factor and its expression was stimulated when cells experienced with starvation, we introduced the neutralizing antibody to block IL6 in HepG2 cells with starvation. The MTT result confirmed that blocking IL6 reversed the resistance of starved cells to sorafenib (Fig. 2g). However, we noticed that when p16 or IL6 were inhibited, unstarved cells, which were not senescent, still showed response to sorafenib (Fig. 2f, g), implying that the role for p16/IL6 pathway in promoting sorafenib resistance was 100% dependent on senescence.

To confirm the hypothesis that senescent cells can impact the cytotoxicity of sorafenib in neighboring non-senescent HCC cells through secreting IL6, parent HepG2 cells and the cells with p16 overexpression were co-cultured in a transwell chamber. The concentration of IL6 in the supernatant of bottom HepG2 cells with p16 overexpression was increased (Fig. 2h) and the parent HepG2 cells in upper chamber became resistant to sorafenib (Fig. 2i). Knock-down of IL6 in cells at the bottom wells attenuated p16 overexpression-induced drug resistance of upper chamber cells, implying that the effect of p16 was IL6 dependent (Fig. S1B).

### Inhibitor of DNA binding 1 (ID1) induces senescence through negative regulation of p16

**Fig. 3:**
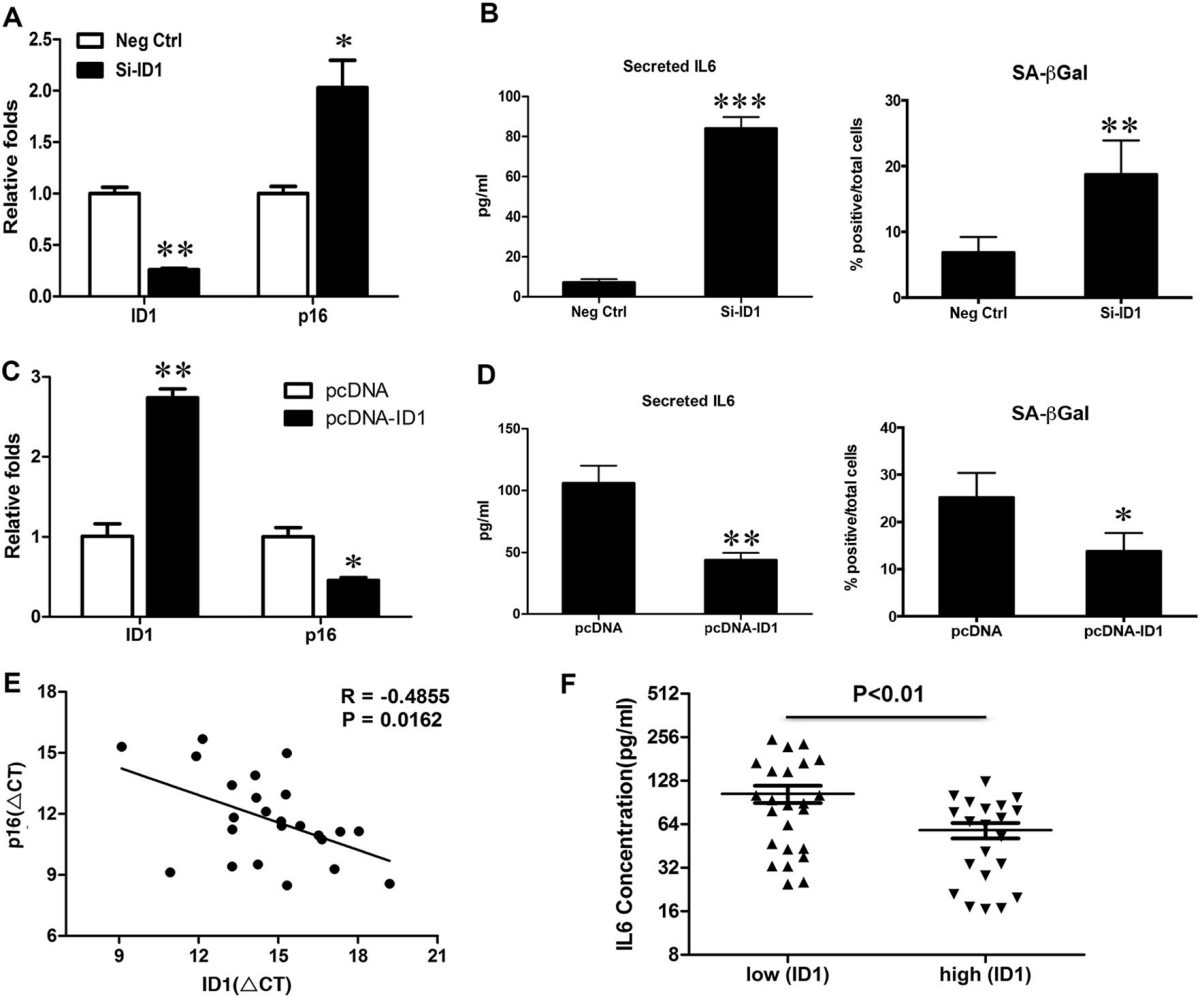
Negative correlation between ID1 and p16/IL6 axis in HCC. **a**, **b** HepG2 cells transfected with siRNA against ID1 (Si-ID1) for 48 h. **a** RNA was isolated to detect ID1 and p16 to evaluate knockdown efficiency and its impact on p16 mRNA expression. **b** IL6 concentrations were quantified by ELISA (left). Percentages of SA-β-gal positive cells were calculated (right). **c**, **d** Hep3B cells transfected with pcDNA-ID1 for 24 h. **c** ID1 overexpression efficiency and its impact on p16 mRNA expression were determined by qRT-PCR. **d** IL6 concentrations were quantified by ELISA (left). Percentages of SA-β-gal positive cells were calculated (right). **e** A negative correlation between ID1 and p16 mRNA expression was observed in 24 HCC patient samples. **f** The levels of IL6, which were measured by ELISA, were higher in HCC patients with low concentrations of ID than those with high ones. *p < 0.05; **p < 0.01; ***p < 0.001, compared with relevant controls (Ctrl) or empty pcDNA

Next, we would like to explore the up-stream pathway of senescence-induced chemo-resistance. We observed that knockdown of ID1 in HepG2 stimulated p16 expression (Fig. 3a), accompanied with the increase of IL6 and SA-β-gal activity (Fig. 3b). However, overexpression of ID1 in Hep3B cells reduced the expression of p16 and IL6, as well as SA-β-gal activity (Fig. 3c, d). To further confirm the relationship between ID1 and p16, their levels were examined in 24 HCC patient samples by qRT-PCR and a negative correlation was found between them (Fig. 3e). We next performed immunohistochemistry to detect the expression of ID1 in 54 HCC samples (Fig. S2A). Negative to weak expression of ID1 was observed in 28 cases (51.9%), which were included in the low level of ID1 group. Moderate to strong expression of ID1 was observed in 26 cases (48.1%), which were included in the high level of ID1 group (Fig. S2B). We analyzed the concentration of IL6 in corresponding blood samples derived from the 54 patients. ELISA experiment indicated that patients with ID1 low level exhibited higher concentrations of IL6, compared with the patients with high ID1 expression (Fig. 3f).

### ID1-p16 axis-mediated sorafenib resistance is IL6-dependent

**Fig. 4:**
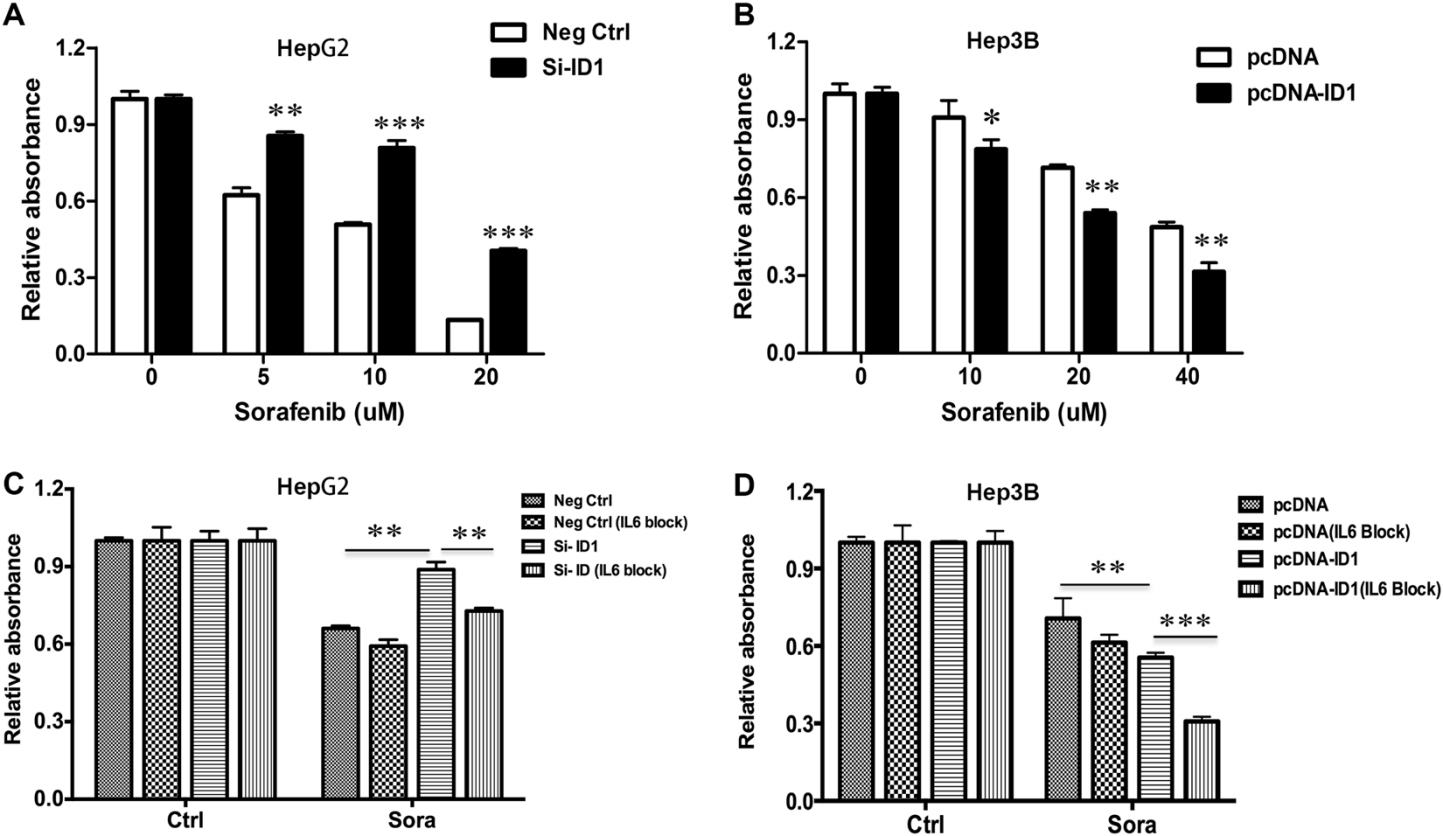
ID1-mediated chemo-resistance. **a** After transfection with si-ID1 for 48 h, MTT assay was employed to detect the efficacy of sorafenib in HepG2 cells with ID1 knockdown. **b** After transfection with pcDNA-ID1 for 24 h, MTT assay was employed to detect the efficacy of sorafenib in Hep3B cells with ID1overexpression. **c** After transfection, HepG2 cells were pretreated with neutralizing antibody against IL6 (5 ng/ml) for 2 h prior to co-treatment with sorafenib (5 μM) for 24 h following by MTT assay. **d** After transfection, Hep3B cells were pretreated with neutralizing antibody against IL6 (5 ng/ml) for 2 h prior to co-treatment with sorafenib (20 μM) for 24 h following by MTT assay. *p < 0.05; **p < 0.01; ***p < 0.001, compared with relevant control (Ctrl)

Our initial observations suggested that ID1 was a key regulator of cellular senescence in HCC. We further asked whether ID1-induced senescence could be used to explain sorafenib resistance. We then detected the expression of ID1 in the above-mentioned five HCC cell lines and found that ID1 was highly expressed in three sorafenib-sensitive cell lines: HepG2, SK-Hep1, and Huh7. In contrast, it was hardly detected in two sorafenib-resistant cell lines, PLC5 and Hep3B (Fig. S2C). Next, we performed the loss-of-function and gain-of-function experiments to further elucidate the role of ID1 in mediating chemotherapy. Forty-eight hours after transfection of the siRNA against ID1, HepG2 cells were treated with increasing concentrations of sorafenib for 24 h. The cells transfected with si-ID1 were less sensitive to sorafenib than cells transfected with negative control (Fig. 4a). However, Hep3B cells transfected with pcDNA-ID1 became sensitive to sorafenib than cells transfected with the empty vector (Fig. 4b).

Our data suggest that IL6 is an important effector in ID1-mediated chemotherapy efficacy. Actually, the anti-apoptotic function of IL6 and its promotion on drug-resistance has already been reported^17^. Tumor cells with high endogenous IL6 were more resistant to drug treatment than those with lower endogenous IL6^17^. We observed that IL6 blocking significantly reduced the resistance of HepG2 cells to sorafenib treatment compared with the cells without IL6 neutralizing antibody (Fig. 4c). Moreover, in Hep3B cells with ID1 overexpression, blocking IL6 increased the response to sorafenib (Fig. 4d). These evidences demonstrated that IL6 was indeed the downstream and critical effector of ID1-mediated chemo-resistance.

### ID1 knockdown contributes to the resistance to sorafenib in vivo

**Fig. 5:**
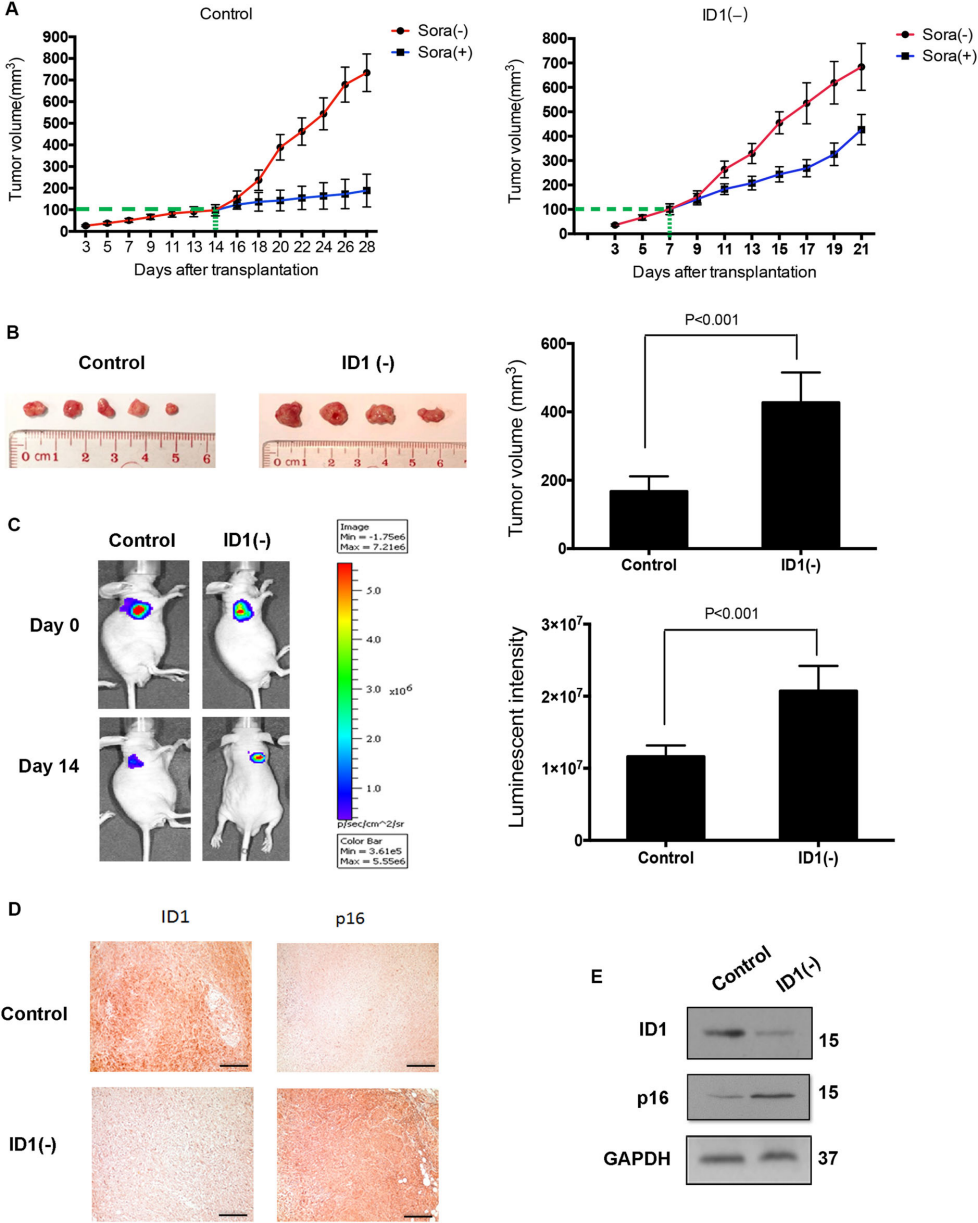
Senescence induced by ID1 knockdown impairs sorafenib efficacy in vivo. All the recipient mice were divided randomly into two groups (*n* = 10 per group). Mice in control group were injected with HepG2-shCtrl cells. Mice in ID1(−) group were injected with HepG2-shID1 cells. Mice were sacrificed after daily administration of sorafenib for 14 days. **a** Curves of tumor growth in control group (left) and ID1(−) group (right). Green lines indicate the time of the beginning of treatment. **b** Morphologies of collected tumors in each group (left). Collected tumor volumes were measured by digital caliper and presented as a histogram (right). **c** Representative mice were monitored by IVIS Imaging System at day 0 and day 14 after sorafenib administration (left). Image intensity was measured and presented as a histogram (right). Tumor tissues were collected for IHC analysis (**d**) and western blot (**e**). The scale bars represent 25 μm. All immunoblots indicate molecular size markers in kDa

We next would like to further confirm the negative effect of ID1-induced senescence on chemotherapy in vivo; we established stable ID1 knockdown cell lines in HepG2 through lentivirus infection, and the nude mouse xenograft assay was employed. Consistent with the known concept that senescent cells can contribute to the growth of non-senescent cancer cells^6,7^, the tumors in mice with ID1(−)-induced senescence grew faster than controls for about 1 week (Fig. 5a). As shown in Fig. 5b, the tumor volume was higher in ID1-knockdown group, indicating resistance to sorafenib. In addition, tumor volumes at day 0 and day 14 after sorafenib administration were monitored using a bioluminescence-based IVIS Imaging System 200 (Fig. 5c, left). The bioluminescent intensities of tumors were plotted as a graph (Fig. 5c, right). Immunohistochemical analysis verified the downregulation of ID1, and p16 was increased in tumors of ID1-knockdown mice (Fig. 5d), accompanied with the enhancement of SA-β-gal activity (Fig. S3). Similar changes in the levels of ID1 and p16 were further confirmed by western blot (Fig. 5e).

### ID1-p16 axis-mediated the secondary resistance to sorafenib in HCC is dependent on IL6/AKT

**Fig. 6:**
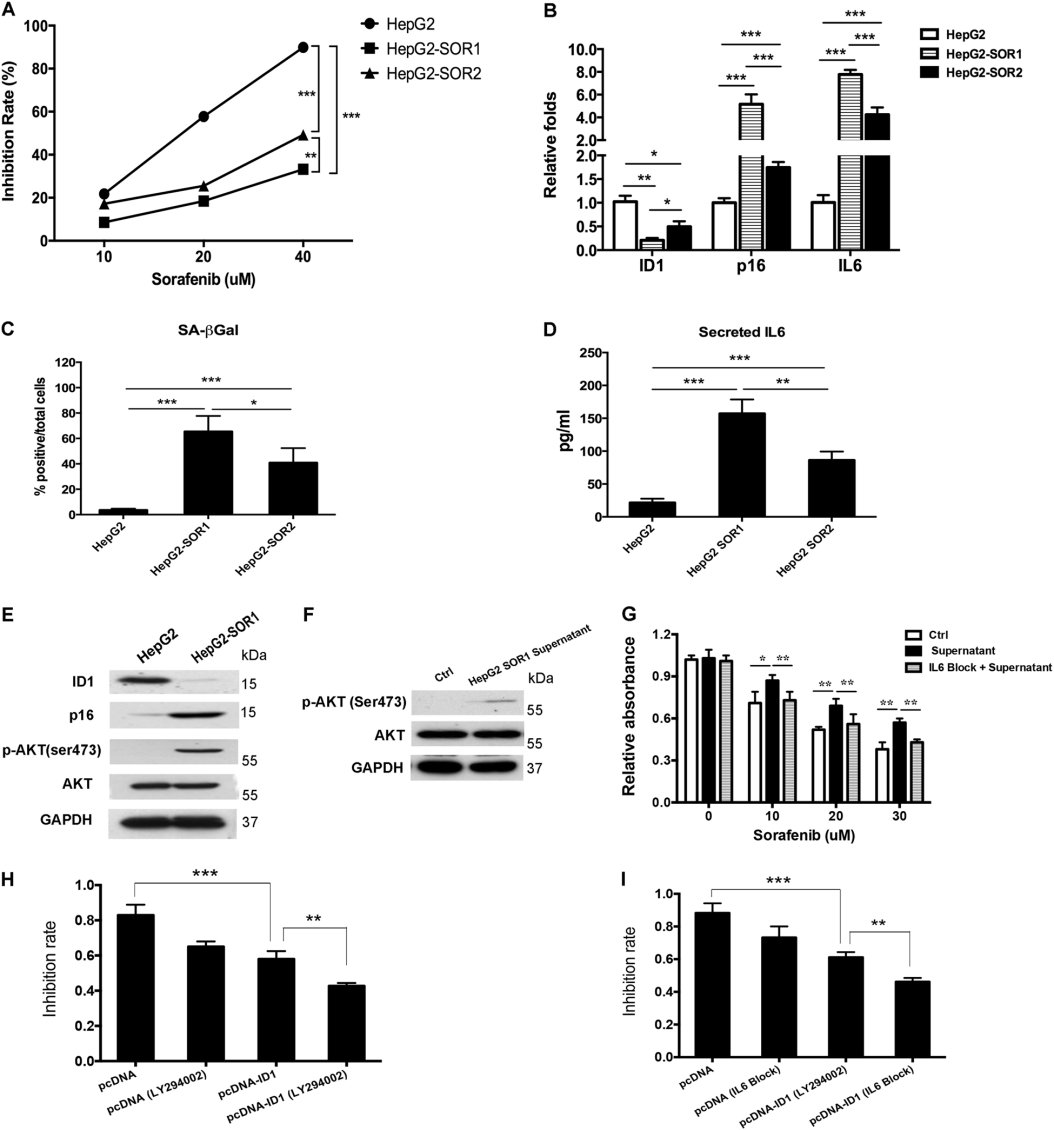
ID1-p16 axis-mediated secondary resistance of sorafenib in HCC is dependent on the activation of IL6/AKT signaling pathway. **a** HepG2-SOR1 and HepG2-SOR2 were incubated with sorafenib for 24 h, MTT assay was employed to observe the efficacy. **b** The expression of ID1, p16, and IL6 mRNA in sorafenib-resistant cell lines was measured by qRT-PCR. **c** The number of positive SA-β-gal stained cells in sorafenib-resistant cell lines was determined. **d** Cells were seeded in 6-well plates and cultured for 24 h. Cell supernatant were collected and the secreted IL6 proteins were quantified by ELISA. **e** Changes of ID1, p16, and p-AKT(473) protein expression in HepG2-SOR1 was detected. **f** HepG2 cells were incubated with the conditioned medium from HepG2-SOR1 for 24 h. An obvious activation of p-AKT(473) was detected. **g** HepG2 cells incubated with the supernatant of HepG2 SOR1 were pre-treated with or without IL6 blocking antibody. The difference of sorafenib efficacy in the cells treated with the supernatant alone or the supernatant plus IL6 blocking was confirmed by MTT assay. **h** 24 h after transfection of pcDNA-ID1 plasmid or empty vector, HepG2 SOR1 cells were pretreated with LY294002 (5 μM) for 1 h prior to co-treatment with sorafenib (20 μM) for 24 h following by MTT assay. Inhibitory rate was analyzed through comparing the average absorbance value in treated cells to control cells. **i** 24 h after transfection of pcDNA-ID1 plasmid or empty vector, HepG2 SOR1 cells were pretreated with neutralizing antibody against IL6 (5 ng/ml) for 2 h prior to co-treatment with sorafenib (20 μM) for 24 h following by MTT assay. Inhibitory rate was analyzed through comparing the average absorbance value in treated cells to control cells. *p < 0.05; **p < 0.01; ***p < 0.001. All immunoblots indicate molecular size markers in kDa

Above data proved that ID1/p16 axis-mediated SASP contributed to the resistance of HCC to sorafenib, both in vitro and in vivo. We also constructed two sorafenib-resistant cell lines, which named HepG2 SOR1 and HepG2 SOR2. Both of these two established cell lines showed less sensitive to sorafenib. According to the MTT assay, HepG2 SOR1 cell was more resistant to sorafenib than HepG2 SOR2 (Fig. 6a). qRT-PCR experiments implied that the ability of resistance to sorafenib was positively correlated with the induction of ID1 but the reduction of p16/IL6, as the ID1 expression was much lower in HepG2 SOR1 than in HepG2 SOR2, accompanied with a higher level of p16 and IL6 (Fig. 6b). In addition, we found that SA-β-gal positive stained cells were increased and IL6 levels in the culture medium were elevated in sorafenib-resistant cell lines (Fig. 6c, d).

Since HepG2 SOR1 was more resistant to sorafenib, we further observed that the resistance was in line with the changes of ID1 and p16 protein expression (Fig. 6e). In addition to the loss of ID1 and stimulation of p16, activation of AKT phosphorylation at ser473 was also observed (Fig. 6e). AKT phosphorylation, which is often activated as a compensatory pathway during acquired resistance, has proved to be constitutively activated in many kinds of drug resistance^18^, including sorafenib^19^. AKT phosphorylation was inhibited in HepG2 SOR1 cells with IL6 blockade. Moreover, a similar result was observed in LY294002-treated HepG2 SOR1 cells (Fig. S4A). We hypothesized that the pro-chemo-resistant role of IL6 was probably due to the activation of AKT phosphorylation. Therefore, we incubated HepG2 cells with the conditioned medium from HepG2 SOR1, in which the IL6 concentration was higher than its parent HepG2 cells. Expectedly, the phosphorylation of AKT at ser473 was stimulated (Fig. 6f). The hypothesis was further confirmed by cell viability assay in which HepG2 cells cultured with conditioned medium from HepG2 SOR1 became resistant to sorafenib (Fig. 6g). Moreover, if HepG2 cells were incubated with IL6 blocking antibodies, the effect of conditioned medium could be counteracted (Fig. 6g). However, we did not observe the occurrence of senescence in HepG2 cells, as the expression of p16 remained unchanged and SA-β-gal staining was still negative or weak (Fig. S4B, C). ID1 up-regulation reversed the resistance of sorafenib in HepG2 SOR1 (Fig. 6h). In order to confirm the point that IL6/AKT signaling pathway may be the downstream effector of ID1-mediated sorafenib resistance, a neutralizing antibody against IL6 and a commonly used PI3K/AKT inhibitor LY294002 were employed in the study. According to the MTT assay, ID1 overexpression-induced reversal of drug resistance was augmented when cells pretreated with LY294002 (Fig. 6h). In addition, we discovered a positive role of IL6 in the development/maintenance of sorafenib resistance in HepG2 SOR1 since a synergistic effect of ID1 overexpression and IL6 blocking on the decrease of the sorafenib resistance was observed (Fig. 6i).

### Sorafenib-induced cellular senescence promotes the development of its acquired resistance

**Fig. 7:**
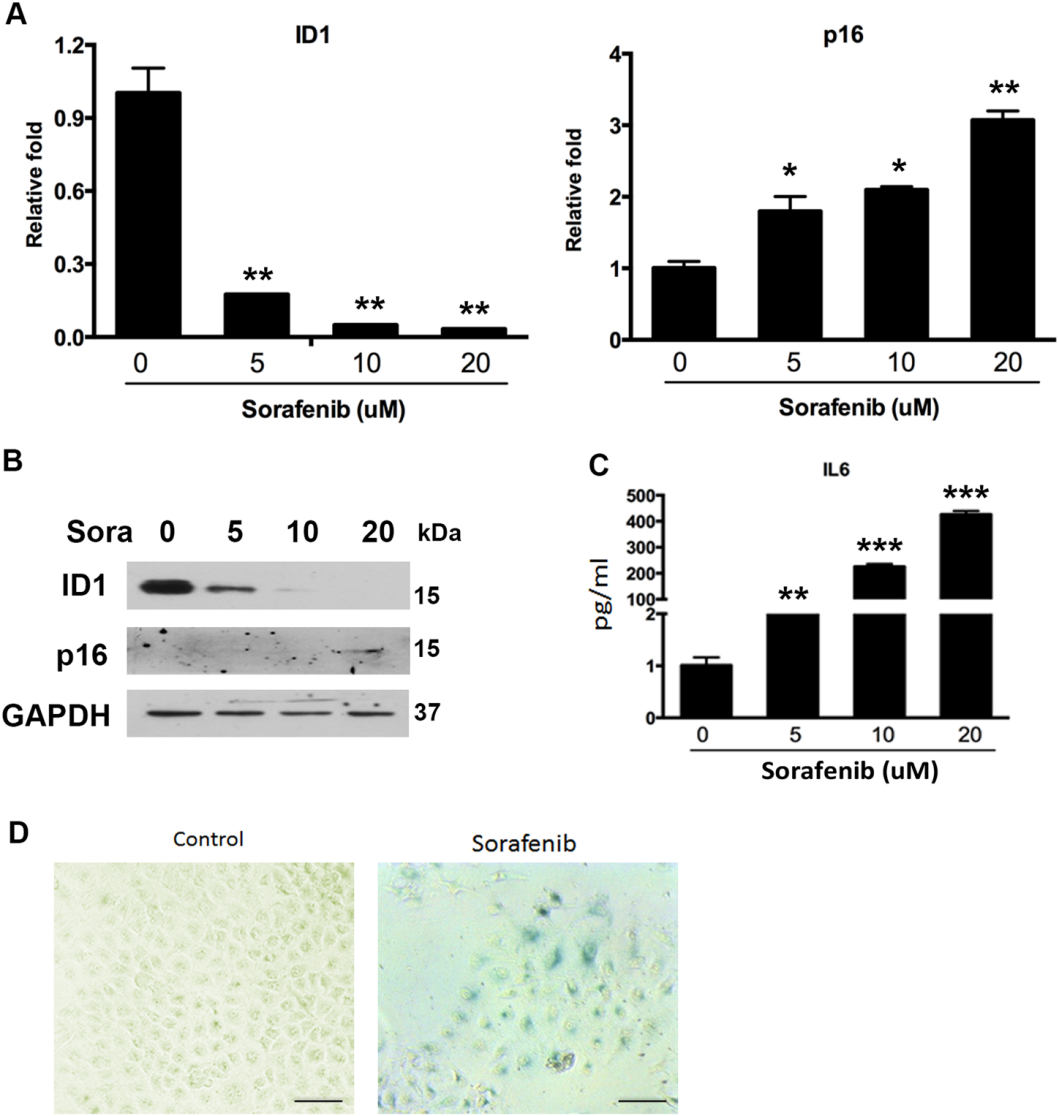
Sorafenib regulates the expression of ID1/p16/IL6. **a** RT-PCR was conducted in HepG2 cells treated with different concentrations of sorafenib for 24 h. **b** Western blot experiments were performed in HepG2 cells treated with different concentrations of sorafenib for 24 h. **c** Cell supernatant was collected to perform the IL6 ELISA assay. **d** After incubated with 5 μM sorafenib for 24 h, cells were stained with SA-β-gal. *p < 0.05; **p < 0.01; ***p < 0.001, compared with Control. The scale bars represent 25 μm. All immunoblots indicate molecular size markers in kDa

In addition to the effect of ID1/p16/IL6 axis on sorafenib efficacy, we observed that sorafenib administration in HCC cells induced senescence through dose-dependently inhibiting ID1 expression but stimulating p16 and IL6 (Fig. 7a–c), accompanied with the enhancement of SA-β-gal activity (Fig. 7d). Similar to the evidence that AKT phosphorylation is activated to protect cells from sorafenib-mediated cytotoxicity^19^, ID1 inhibition and subsequent p16/IL6 activation were supposed to be, in this study, a cytoprotective mechanism to resist sorafenib-induced cell death. This point is in line with the result from Fig. 4 that overexpressing ID1 enhanced the anti-tumor effect of sorafenib in resistant Hep3B cells. We hence concluded that this mechanism contributed to the development of acquired/secondary resistance.

## Discussion

Clinical trials identified sorafenib as the standard of chemotherapy for advanced HCC. However, the low clinical response rate has limited its application^20,21^. We hold an opinion that the acquisition of sorafenib resistance is a result of intricate process involved in different molecular and cellular factors^22^. The phenomenon that some HCC cells are initially resistant to sorafenib is termed primary resistance. Consistent with previous reports that primary resistance of HCC to sorafenib resulted from genetic heterogeneity, such as EGFR activation^23^, and HBV expression^24^, in our study, five HCC cell lines with different genetic backgrounds exhibited different sensitivities to sorafenib. Moreover, in sorafenib-responsive HCC patients, acquired/secondary resistance is unavoidable after long-term exposure to sorafenib. Our study has demonstrated that SASP is involved in both the primary and secondary resistance of sorafenib in HCC. HCC cells with a high level of SASP, which comes true by the negative effect of ID1 on p16 as well as IL6, exhibited an obvious resistance to sorafenib. HCC cell lines are highly heterogenetic. We have to admit that our discoveries, which mainly based on two typical cell lines HepG2 and Hep3B, may not be applicable to all subtypes of HCC cells. In addition, the regulation of senescence is complicated and involved in different molecules and pathways. Though our work has uncovered ID1 as a potential regulator in the process of p16-mediated senescence, the role of other well-known regulators, such as p53, are still unclear. As previously reported, p53 promotes senescence through activating the transcription of relevant genes, such as p21 and PML, PML in turn recruits p16^25^. The potential role of p53 in this study is not clear but will be explored in future work.

At present, there is a debate about the role of cellular senescence in cancer, especially in chemotherapy^26–29^. As discussed by Lecot and his colleagues, which direction, beneficial or detrimental, plays a dominant role for senescence in cancer treatment, is dependent on the context of cancer type and what kinds of secretory factors accumulated in tumor microenvironment^28^. Our study supports the pro-tumorigenic role of senescence in HCC due to its contribution to chemoresistance. However, the detailed mechanism remains unclear. Further work is required in this direction to explore the most important pathways or senescence-related gene mutations that are responsible for the malignant phenotype. Exploring the genetic/epigenetic alterations that occurred in HepG2 SOR1/2 lines will also be our further study in the future.

We observed that sorafenib administration in HCC cells could induce senescence through its regulation on ID1/p16/IL6 axis, which is parallel to the induction of cell apoptosis and inhibition of cell proliferation. It has been reported that some chemotherapeutic agents can promote cellular senescence^30,31^. In the present study, we speculate that the acquirement of secondary resistance may rely on the continuous long-term activation of ID1/p16/IL6 axis, which in turn contributes to the switch of HCC cell lines from sensitive to resistant to sorafenib. This point, which is shown in a scheme but needs to be explored deeply (Fig. S5), provides a theoretical evidence for our future further studies.

Our current observations both confirm and argue against some of the existing knowledge regarding the status and function of ID1 in HCC. In our study, we observed ID1 protein was down-regulated in 15 out 20 HCC tumors compared to matched non-tumor tissues (Fig. S2D). This result is consistent with Damdinsuren et al.'s report, in which ID1 protein was proved to be highly expressed in non-tumor liver tissues with hepatitis and cirrhosis. Moreover, analysis of the Cancer Genome Atlas (TCGA) RNA-seq dataset further confirmed the decreased expression of ID1 in 372 liver HCC samples, compared to adjacent normal samples (Fig. S6). To our knowledge, both good and harmful prognostic significance for ID1 in HCC have been observed, but no studies focus on its function for HCC chemotherapy yet^32,33^. The anti-chemo-resistant role of ID1 has been revealed in prostate cancer and lung cancer. In our study, we identified ID1 as a contributor to overcome sorafenib resistance through mediating senescence. Combined with ID1 overexpression, sorafenib administration in HCC cells exhibited an increased cytotoxicity.

Collectively, our study demonstrated that SASP-related p16/IL6 axis is responsible for sorafenib resistance, providing a new strategy for HCC patients to overcome the acquisition of sorafenib resistance. However, we have to admit that it would be better to confirm our discoveries through clinical observation.

## Materials and methods

### Reagents and antibodies

Sorafenib (Cat No: S-8502) was purchased from LC Laboratories. LY294002 (Cat No: L9908) and MTT (Cat No: M2003) were obtained from Sigma-Aldrich. ID1 overexpression plasmid pcDNA3 hId1(Cat No: #16061) and p16 overexpression plasmid pCMV p16 INK4A (Cat No: #10916) were purchased from Addgene. Antibodies against p-AKT (ser473) (Cat No: #4060) and p16 (Cat No: #2407) were purchased from Cell Signaling Technology. Antibodies against ID1 (Cat No: sc-488) and GAPDH (Cat No: sc-47724) were purchased from Santa Cruz Biotechnology. Neutralizing antibody of IL6 (Cat No: MAB206) was purchased from R&D Systems.

### Cell lines and cultures

The HCC cell lines PLC/PRF/5 (PLC5), HepG2, Hep3B, SK-Hep1, and Huh7 were purchased from ATCC and stored at liquid nitrogen in our lab. All these cells were cultured in DMEM medium supplemented with 10% fetal bovine serum (Invitrogen).

### Cell viability and apoptosis assay

Cells were seeded into 96-well plates and treated with different concentrations of sorafenib. MTT assay was employed to measure the cell viability. The half inhibitory concentration (IC50) value was determined for each cell lines. The inhibition rate (%) was calculated as follows: (1 − survival cells/control)% = (1 − the mean value of absorbance in treated group/the mean value of absorbance in control group)%. In transwell co-culture system, HepG2 cells were seeded in the bottom wells of the 24-well plates and transfected with pCMV-p16 or pcDNA-3.1. After 4–6 h, medium was changed with DMEM containing 10% FBS. Upper chamber with pre-seeded normal HepG2 cells was inserted and co-cultured for 48 h. Then, HepG2 cells in upper chamber were incubated with different concentrations of sorafenib for 24 h, cell viability was determined by MTT assay.

### Senescence-associated β-galactosidase (SA-β-gal) staining

The detection of SA-β-gal activity was performed with Senescence β-Galactosidase Staining Kit (Cell Signaling Technology, Cat No: #9860) according to the provided protocol. Cells seeded in 6-well plates were incubated with freshly prepared β-gal staining solution (pH 6.0) at 37 °C overnight. Percentages of SA-β-Gal positive cells in each well were calculated by counting the number of blue cells in six fields (200× total magnification).

### Enzyme-linked immunosorbent assay (ELISA)

IL6 concentration in supernatants collected from cultured cells that seeded in 6-well plates was measured with the human quantitative IL6 ELISA kit (R&D Systems, Cat No: #6050) and normalized to the cell numbers. After being thawed on ice and centrifuged at 4 °C for 20 min, 50 μl serum samples collected from HCC patients were used to perform ELISA assay according to the manufacturer’s instruction.

### Real-time quantitative PCR (RT-qPCR)

Total RNA was isolated using TRIzol Reagent (Invitrogen) according to the manufacturer’s instructions. The RNA yield and A260/280 ratio were determined by a NanoDrop spectrophotometer (Thermo Fisher Scientific). 1 μg of total RNA from each sample was reverse-transcribed into cDNA with PrimeScript^TM^ 1st Strand cDNA Synthesis Kit (Takara). 50 ng cDNA was used as a template for real-time PCR. The primers used for PCR were as follows: ID1-F AATCCGAAGTTGGAACCCCC, ID1-R ACACAAGATGCGATCGTCCG; p16-F ATGGAGCCTTCGGCTGACT, p16-R ACCGTAACTATTCGGTGCGT; IL6-F GACCCAACCACAAATGCCAG, IL6-R GCTGCGCAGAATGAGATGAG; GAPDH-F TCAAGGCTGAGAACGGGAAG, GAPDH-R TCGCCCCACTTGATTTTGGA.

### Tissue specimens

Specimens, including paraffin-embedded sections, frozen tumor tissues, and serum samples were collected from HCC patients who underwent initial treatments at the Prince of Wales Hospital from January 2007 to January 2010. Patient information is summarized in Supplementary Table 1. Written informed consent was received from all patients for subsequent use of their resected tissues. Paraffin-embedded sections were used to detect the protein expression of ID1 by immunohistochemistry. Total RNA was extracted from frozen tumor tissues to perform RT-qPCR. The concentrations of IL6 in blood samples collected from HCC patients were determined by ELISA.

### Western blot

Cells and tissues were lysed with RIPA buffer in the presence of Protease Inhibitor Cocktail (Pierce, Rockford, USA). Protein concentration was measured using a BCA Protein Assay Kit (Pierce, Rockford, IL). The experiment was performed as previously reported^34^.

### Immunohistochemistry (IHC)

Tumor specimens from HCC patients and mouse tumors were fixed overnight in formalin and processed into 5 μm thick sections. The immunohistochemistry of ID1 and p16 was performed as previously described^35^. Staining intensity was graded as follows: negative, weak, moderate, and strong.

### Construction of stable ID1-knockdown HepG2 cell line

HepG2 cells with stable ID1-knockdown were obtained through the transfection of lentiviral particles containing ID1-targeted short hairpin RNA (shRNA). A ID1-targeted shRNA sequence and a scrambled sequence were inserted into the linearized pLKO.1 expression vector to construct the pLKO-shID1 and pLKO-shCtrl vector, respectively. They were then co-transfected into 293T cells with lentiviral helper plasmids psPAX2 and pMD2G using Lipofectamine 2000 (Invitrogen) according to the protocols provided by Addgene. The sequence of shRNA oligos of ID1 and negative control were as follows (target sequence was underlined): ID1 (forward) 5′-CCGGATCGCATCTTGTGTCGCTGAACTCGAGTTCAGCGACACAAGATGCGATTTTTTG-3′; ID1 (reverse) 5′-AATTCAAAAAATCGCATCTTGTGTCGCTGAACTCGAGTTCAGCGACACAAGATGCGAT-3′ Negative ctrl (forward) 5′-CCGGCAACAAGATGAAGAGCACCAACTCGAGTTGGTGCTCTTCATCTTGTTGTTTTTG-3′; Negative ctrl (reverse) 5′-AATTCAAAAA CAACAAGATGAAGAGCACCAACTCGAGTTGGTGCTCTTCATCTTGTTG-3′. The knockdown efficacy of ID1 was confirmed by western blot 5 days after transfection.

### ID1/p16 overexpression and small interference RNA (siRNA) treatment

Cells were seeded in 6-well plates. Plasmid was transfected into cells for 24 h using X-tremeGENE HP DNA Transfection Reagent (Roche). Chemically synthesized siRNAs targeting ID1 (sc-29356) or p16 (sc-37622) were purchased from Santa Cruz Biotechnology and respectively transfected into HepG2 or Hep3B cells for 48 h using Lipofectamine® 2000 (Invitrogen).

### Generation of sorafenib-resistant cell line

Initially, 1 × 10^4^ HepG2 cells in 6-well plate were incubated with 5 μM sorafenib for 48 h. Dead cells were washed out with PBS. After the cells were recovered to normal growth and proliferation, survival cells were continuously treated with increased concentrations of sorafenib (0.5 μM per time). Over several months, we developed two HepG2 cell lines that were resistant to sorafenib (HepG2-SOR1 and HepG2-SOR2). After establishment, resistant cell lines were maintained in medium with 1 μM sorafenib.

### Tumor xenograft assay

4–6 weeks-old male nude mice were provided by the Laboratory Animal Services Centre of our institute. Mice were divided randomly into two groups (*n* = 10 per group) and injected with 2 × 106 cells/100 μl of HepG2-shCtrl and HepG2-shID1 cells, respectively. The injected cells stably expressed luciferase. Tumor volume was measured by caliper and calculated with the formula: (length × width^2^)/2. To observe the different efficacy of sorafenib between ID1(−) group and control group, sorafenib was administrated in the two groups of mice with equal size of tumors (100 mm^3^). As tumor growth was fast in ID1(−) group than in control group for about one week, the start time of treatment was different in the two groups. Through random selection, half of mice in each group were received sorafenib treatment and another half of mice were without the treatment. ID1(−) mice (*n* = 5) was treated at day 7 after transplantation, and control mice (*n* = 5) was treated at day 14 after transplantation. Both of the two groups received drug treatment for 14 days. At the end, we stopped the experiment at day 21 after transplantation for ID1(−) mice, and day 28 after transplantation for control mice. One ID1(−) mice in treatment group was dead before day 28 after transplantation. All the mice were sacrificed and tumors were collected. Tumor tissues were subjected to IHC staining and SA-β-gal staining. Tissue lysates were analyzed by Western blotting to detect the expression of ID1 and p16. Sorafenib was dissolved in Cremophor EL/ethanol 50:50 at fourfold the dose. Equal volume of Cremophor EL/ethanol solution served as the control vehicle. After completely dissolved, the solution was diluted with water to the required dose. To monitor the response of tumor-bearing mice to sorafenib, mice were anesthetized with isoflurane, given a single i.p. dose of 150 mg/kg D-luciferin in PBS and imaged 8 min after injection (IVIS Spectrum, Caliper Sciences). Results were analyzed using Living Image software. All animal procedures were approved by the Animal Experimentation Ethics Committee of the Chinese University of Hong Kong and are in accordance with the Department of Health (Hong Kong) guidelines in Care and Use of Animals.

### Statistical analysis

Data are shown as mean ± SD. Statistical differences between the two groups were examined by Student’s *t*-test. *p* Values of ≤0.05 were considered statistically significant.

## Electronic supplementary material

Supplemental Materials

Supplementary figure legends
