# Supplementary material for: ID1-induced p16/IL6 axis activation contributes to the resistant of hepatocellular carcinoma cells to sorafenib
Source: Cell Death Dis. 2018 Aug 28;9(9):852. doi: 10.1038/s41419-018-0926-x (PMC6113298; doi:10.1038/s41419-018-0926-x)
Supplement: Supplementary file 1 — Supplemental Materials [file 41419_2018_926_MOESM1_ESM.docx]

**Supplementary Table and Figures.**

**Table S1.** Clinicopathological characteristics of 54 HCC patients.

| Parameter | Subtype | Patients *n* (%) |
| --- | --- | --- |
| Age (years) | >58 | 25 (46.3) |
|  | ≤58 | 29 (53.7) |
| Gender | male | 43 (79.6) |
|  | female | 11 (20.4) |
| AFP (ug/L) | >500 | 12 (22.2) |
|  | ≤500 | 42 (77.8) |
| HBsAg | negative | 14 (25.9) |
|  | positive | 40 (74.1) |
| Tumor size (cm) | >4.75 | 18 (33.3) |
|  | ≤4.75 | 36 (66.7) |
| Vascular invasion | yes | 11 (20.4) |
|  | no | 43 (79.6) |
| Pathological grade | well | 41 (75.9) |
|  | moderate | 10 (18.5) |
|  | poor | 3 (5.6) |
| AJCC staging | I | 38 (70.4) |
|  | II | 11 (20.4) |
|  | III | 5 (9.2) |

**Figure S1.** p16-induced sorafenib resistance is IL6-dependent. A. The efficiency of p16 after cell transfection with pCMV-p16 and si-p16 was observed by western blot. B. In the transwell co-culture system, HepG2 cells that at the bottom of wells were transfected with si-IL6 and pCMV-p16, the cytotoxicity of sorafenib in the upper chamber of HepG2 cells were detected by MTT assay. *, p<0.05; **, p<0.01; ***, p<0.001, compared with Control.


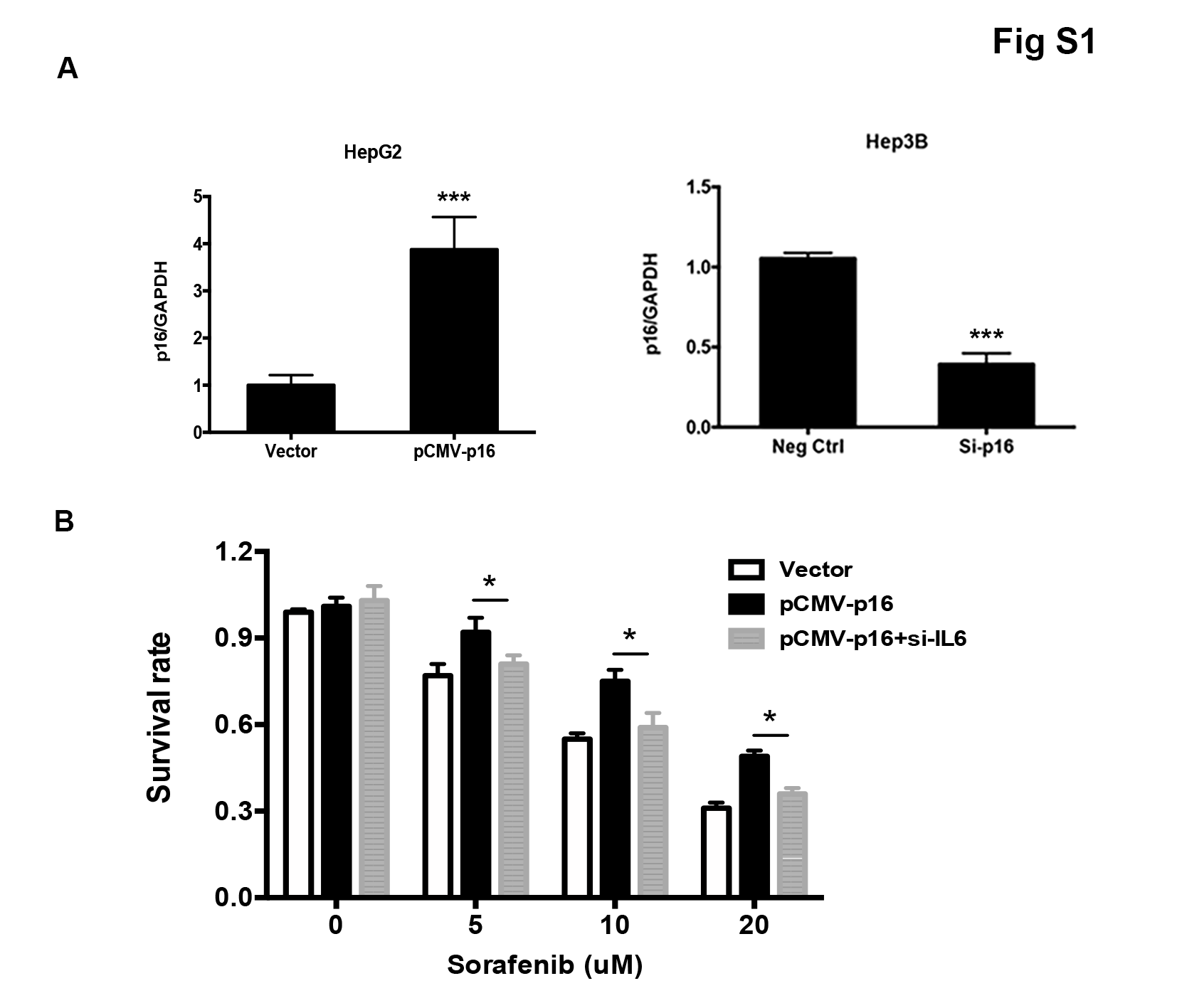


**Figure S2.** Expression of ID1 in HCC. A. Representative images of IHC staining for ID1. B. Statistics of ID1 expression in 54 HCC patient samples. C. Expression of ID1 protein in 20 pairs of HCC tumor tissues and corresponding non-tumor tissues was determined by western blot. (N, non-tumor; T, tumor) D. The levels of ID1 protein in five HCC cell lines were determined by western blot.


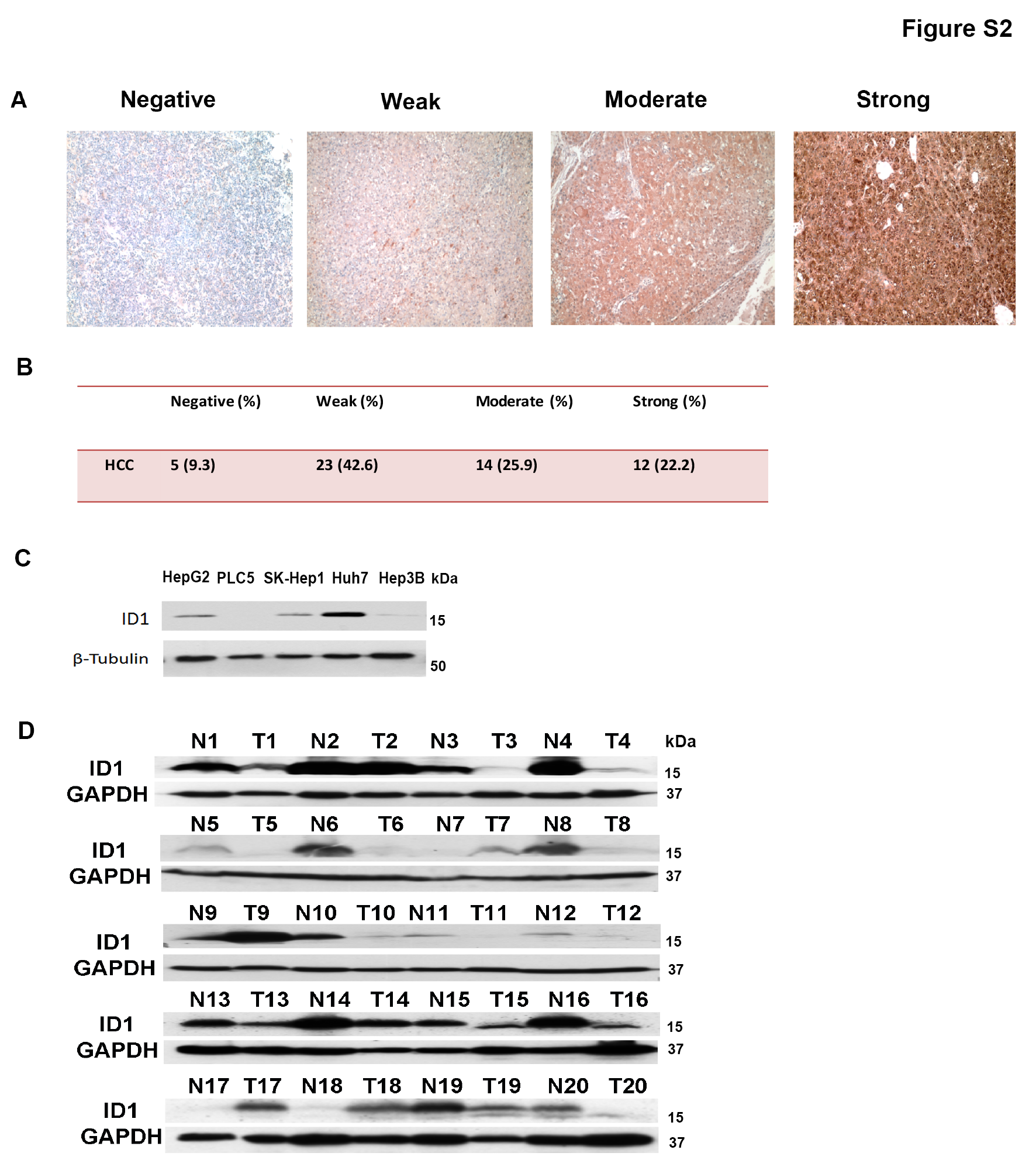


**
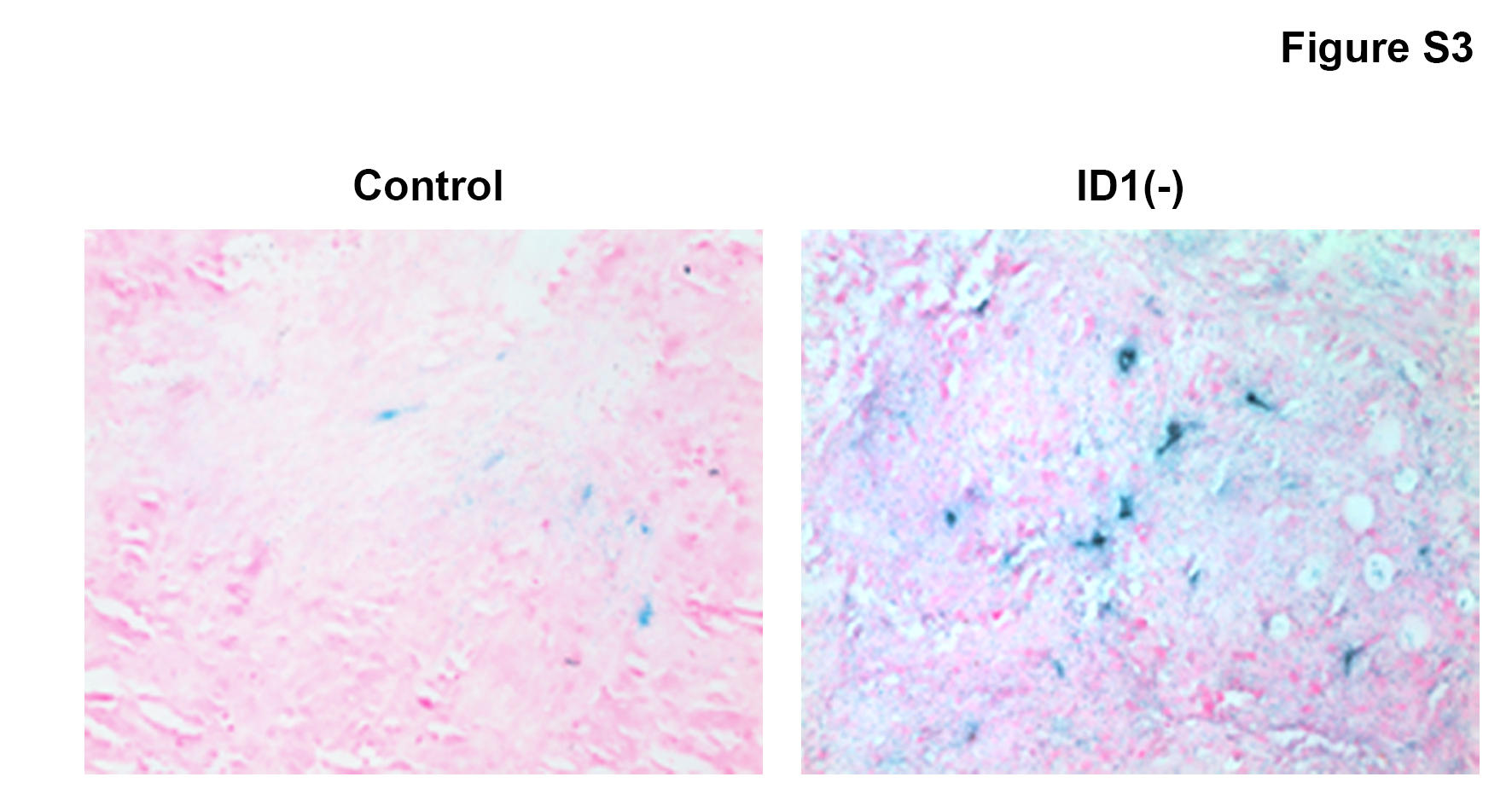
Figure S3.** Tumor tissues collected from nude mice were examined for SA-β activity (blue staining).

**Figure S4.** IL6/AKT axis activation is responsible for the acquired resistance of sorafenib in HCC. A. The expression of p-AKT in HepG2 SOR1 cells with LY294002 incubation or IL6 blocking was examined by western blot. B. HepG2 cells that incubated with the supernatant of HepG2 SOR1 were pre-treated with IL6 blocking antibody. C. The difference of sorafenib efficacy in supernatant alone or combined with IL6 blocking was confirmed by MTT assay.


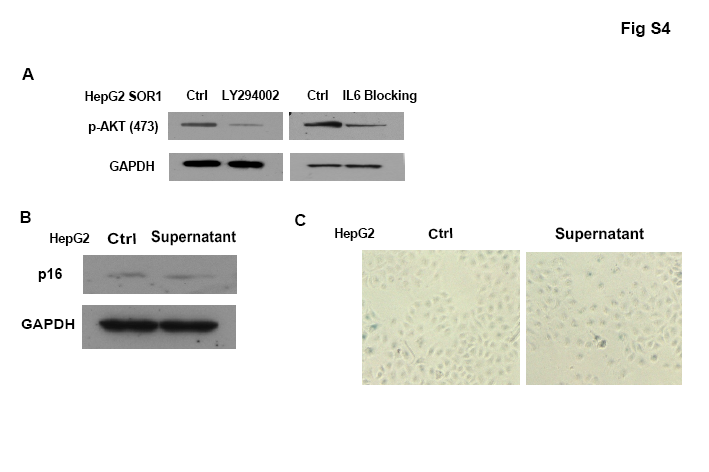


**Figure S5.** Scheme representing the acquired sorafenib resistance. A. Short-term exposure to sorafenib induces cell death of sensitive cells. B. Long-term exposure of sensitive cells to sorafenib induces acquired resistance through accumulative stimulation of SASP.


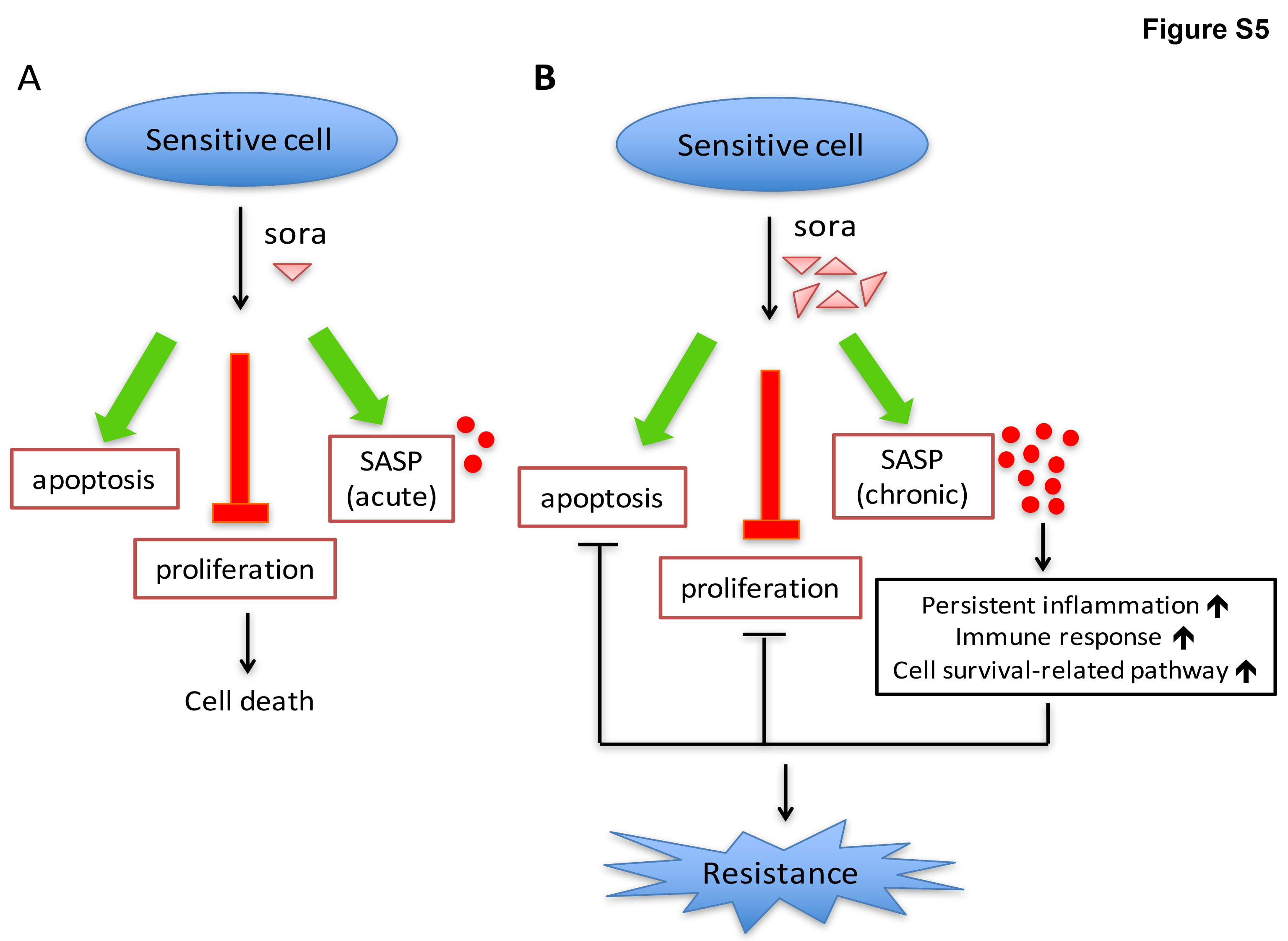


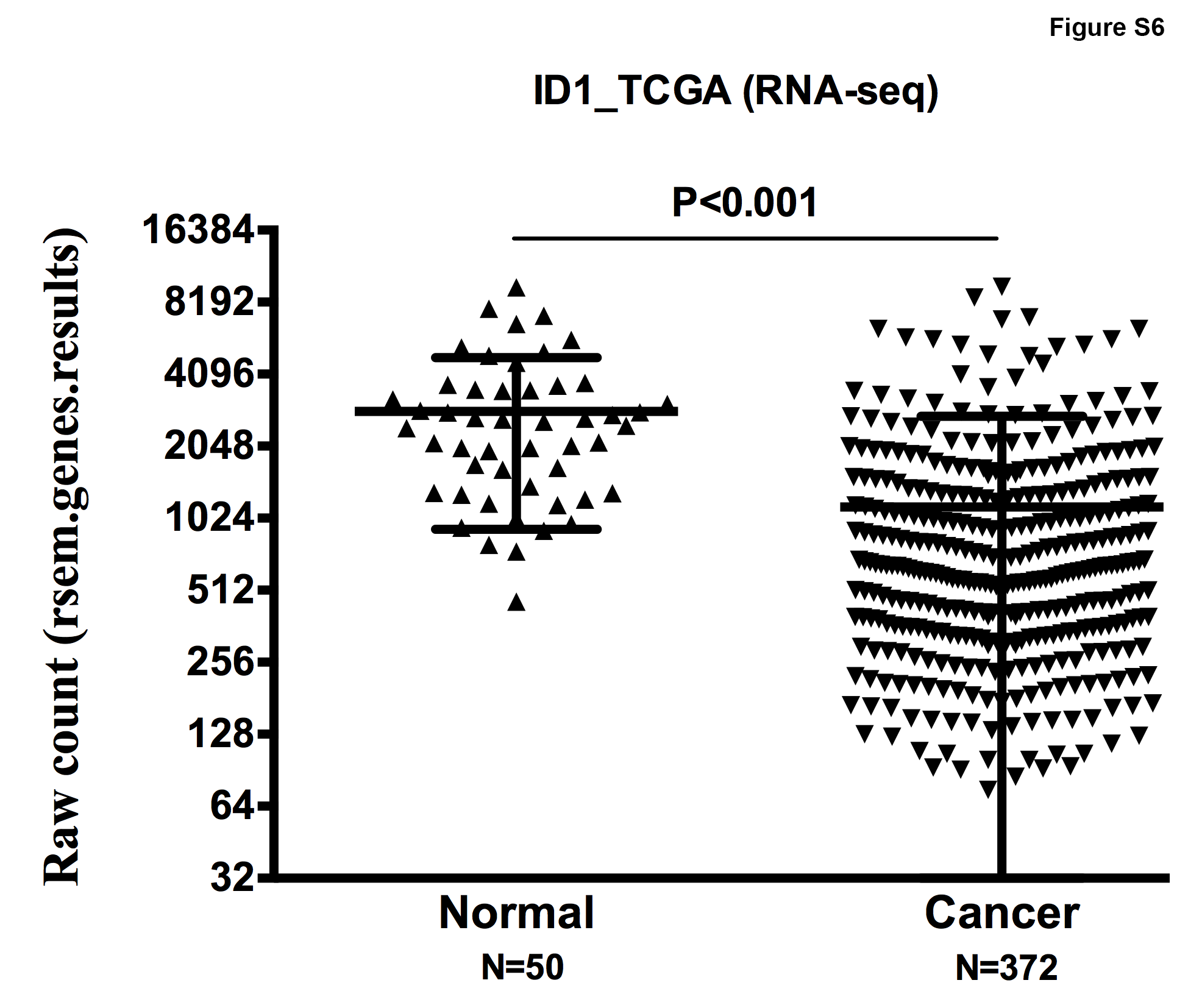
**Figure S6.** The comparison of ID1 expression between cancer tissues and adjacent normal tissues. The raw count of ID1 RNA-seq data was retrieved from TCGA.
