# Supplementary material for: ID1-induced p16/IL6 axis activation contributes to the resistant of hepatocellular carcinoma cells to sorafenib
Source: Cell Death Dis. 2018 Aug 28;9(9):852. doi: 10.1038/s41419-018-0926-x (PMC6113298; doi:10.1038/s41419-018-0926-x)
Supplement: Supplementary file 2 — Supplementary figure legends [file 41419_2018_926_MOESM2_ESM.docx]

**ID1-induced p16/IL6 axis activation contributes to the resistant of hepatocellular carcinoma cells to sorafenib**

Lei-lei Niu^1,2^, Chuan-le Cheng^4^, Ming-Yue Li^2,7^, Sheng-li Yang^2,5^, Bao-guang Hu^2,6^, Charing CN Chong^2^, Stephen L Chan^3^, Jianwei Ren^2^, George G. Chen^2,7*^, Paul B. S. Lai^2*^

**Table S1**. Clinicopathological characteristics of 54 HCC patients.

**Figure S1**. p16-induced sorafenib resistance is IL6-dependent. A. The efficiency of p16 after cell transfection with pCMV-p16 and si-p16 was observed by western blot. B. In the transwell co-culture system, HepG2 cells that at the bottom of wells were transfected with si-IL6 and pCMV-p16, the cytotoxicity of sorafenib in the upper chamber of HepG2 cells were detected by MTT assay. *, p<0.05; **, p<0.01; ***, p<0.001, compared with Control.

**Figure S2**. Expression of ID1 in HCC. A. Representative images of IHC staining for ID1. B. Statistics of ID1 expression in 54 HCC patient samples. C. The levels of ID1 protein in five HCC cell lines were determined by western blot. D. Expression of ID1 protein in 20 pairs of HCC tumor tissues and corresponding non-tumor tissues was determined by western blot. (N, non-tumor; T, tumor).

**Figure S3**. Tumor tissues collected from nude mice were examined for SA-β-gal activity (blue staining).

**Figure S4**. IL6/AKT axis activation is responsible for the acquired resistance of sorafenib in HCC. A. The expression of p-AKT in HepG2 SOR1 cells with LY294002 incubation or IL6 blocking was examined by western blot. B. Supernatants from HepG2-SOR1 were collected to incubate with HepG2, p16 expression was detected by western blot. C. HepG2 cells that treated with HepG2-SOR1 supernatants were incubated with β-gal staining solution.

**Figure S5**. Scheme representing the acquired sorafenib resistance. A. Short-term exposure to sorafenib induces cell death of sensitive cells. B. Long-term exposure of sensitive cells to sorafenib induces acquired resistance through accumulative stimulation of SASP.

**Figure S6**. The comparison of ID1 expression between cancer tissues and adjacent normal tissues. The raw count of ID1 RNA-seq data was retrieved from TCGA.
